# Iterative Refinement of a Sensory-Based Mindfulness Coaching Approach: An Exploratory Qualitative Action Research Study with Korean University Students

**DOI:** 10.3390/bs16091598

**Published:** 2026-09-08

**Authors:** Myoung Jin Hong, Song Yi Lee

**Affiliations:** Department of Counselling and Coaching, Dongguk University, Seoul 04620, Republic of Korea; mindlantern21@gmail.com

**Keywords:** sensory-based mindfulness coaching, sensory awareness, university students, qualitative action research, coaching

## Abstract

This exploratory qualitative action research study examined the implementation and iterative refinement of mindfulness–flow–awareness–coaching (MFAC), a sensory-based mindfulness coaching approach, and explored participants’ reported experiences. Six Korean university students completed pre-MFAC and post-MFAC interviews and the six-session MFAC framework. One participant’s initial Session 4 was discontinued after an unexpected recollection resulted in emotional overwhelm, and an adjusted Session 4 was conducted one week later, resulting in 37 session deliveries. The study drew on interview and session transcripts, participant records, action records, and observational and reflexive notes. The first author conducted a chronological implementation analysis and reflexive thematic analysis, which identified four practice-based principles: prioritising sensory noticing over interpretation, establishing psychological safety before task completion, balancing structural consistency with responsive adaptation, and framing action-oriented activities as revisable attempts. Three themes characterised participants’ reported experiences: greater openness to sensory and emotional experience, contextualised self-understanding through meaning-making, and movement towards feasible action. Participants also reported discomfort, interruptions, ambiguous experiences, and incomplete actions. These findings offer a contextually grounded account of how we refined MFAC and how participants interpreted their engagement. This study positions MFAC as a practice-informed approach rather than as evidence of therapeutic efficacy or causal psychological change. Future research employing diverse methodologies should examine its applicability and transferability to broader populations and settings.

## 1. Introduction

University students in emerging adulthood make important decisions concerning education, careers, relationships, and independence while responding to academic demands, social expectations, and uncertainty about their future (Arnett, 2000; Schwartz et al., 2005). Career uncertainty, social anxiety, perfectionistic self-evaluation, and reliance on external standards may make it difficult for students to recognise their needs, understand their experiences, and make personally meaningful choices (Jang & Choi, 2023; Lee & Kang, 2020). Therefore, support for this population needs to address psychological distress and how students become aware of their present condition, interpret their experiences, and identify feasible actions within their life contexts.

Mindfulness involves attending to present-moment sensations, emotions, and thoughts with less immediate judgment (Bishop et al., 2004; Kabat-Zinn, 2003). Researchers have examined mindfulness-based approaches in relation to stress, anxiety, depression, and well-being (Goyal et al., 2014; Khoury et al., 2013). However, outcome-focused research provides limited understanding of how participants experience mindfulness activities and connect momentary awareness with concerns in their everyday lives. Moreover, researchers and practitioners should not assume that mindfulness is uniformly helpful or use it to frame academic, relational, and economic pressures solely as matters of individual acceptance (Purser, 2019; Van Dam et al., 2018).

Sensory awareness may provide a concrete starting point for participants who find abstract introspection or immediate verbal expression difficult. Interoception contributes to emotional experience and self-awareness (Craig, 2009; Farb et al., 2015), whereas embodied cognition emphasises how interactions among the body, action, and environment shape meaning (Wilson, 2002). By attending to touch, visual form, scent, sound, and taste, participants may notice bodily and emotional responses before evaluating or explaining them. However, sensory experience has no fixed meaning and may evoke discomfort, ambiguity, or limited sensation, as well as engagement. Participants interpret these experiences in relation to their personal history and present context.

Sensory awareness alone does not necessarily lead to contextualised self-understanding or feasible actions. Coaching provides a collaborative dialogue through which individuals can examine their current situations, clarify personally relevant directions, explore alternatives, and select possible actions (Grant, 2003; Grant & Cavanagh, 2007). The GROW framework organises coaching dialogue around goal, reality, options, and will (or way forward) (Whitmore, 2017). Integrating sensory mindfulness with GROW-informed dialogue may therefore provide a structured context in which participants can relate present-moment experiences to their concerns and consider actions that are appropriate to their circumstances. Within this process, participants and coaches co-construct meaning through the coaching relationship, rather than attributing outcomes solely to mindfulness practice.

Previous studies have integrated mindfulness and coaching in educational, developmental, and health-related settings (Collard & Walsh, 2008; Corti & Gelati, 2020; Spence et al., 2008; van den Assem & Passmore, 2022). However, limited research has examined how practitioners implement sensory-based mindfulness and coaching dialogue together, revise practice in response to participants’ sensory and emotional responses or understand participants’ descriptions of their experiences with this process (Hong, 2025). Examining these processes is important because sensory engagement may involve discomfort, ambiguity, or limited sensation, while action planning may create additional burdens when it does not reflect participants’ circumstances.

MFAC’s distinctive contribution is not the introduction of a new mindfulness technique or evidence of superior outcomes. Rather, it offers a practice-informed framework that connects sensory noticing with GROW-informed dialogue for contextual meaning-making and participant-selected, revisable action. Its practical contribution is to make explicit how this core sequence can be maintained while the delivery of sensory activities, dialogue, and action planning is responsively adapted to participants’ needs.

This exploratory qualitative action research study examined the implementation and iterative refinement of mindfulness–flow–awareness–coaching (MFAC), a sensory-based mindfulness coaching approach, and explored Korean university students’ experiences of participation.

This study addresses the following research questions (RQ):

**RQ1.**  *How was MFAC implemented and iteratively refined?*

**RQ2.**  *How did participants describe their experiences of MFAC?*

## 2. Materials and Methods

### 2.1. Qualitative Action Research Design

This study used an exploratory qualitative action research design to examine the implementation and iterative refinement of MFAC and participants’ descriptions of their experiences. Consistent with action research, the first author implemented MFAC through iterative cycles of planning, acting, observing, and reflecting; observations and reflections from earlier deliveries informed subsequent implementation (Kemmis & McTaggart, 2005). Participant experiences were understood as contextual and co-constructed through interaction with the researcher-facilitator.

**Figure 1:**
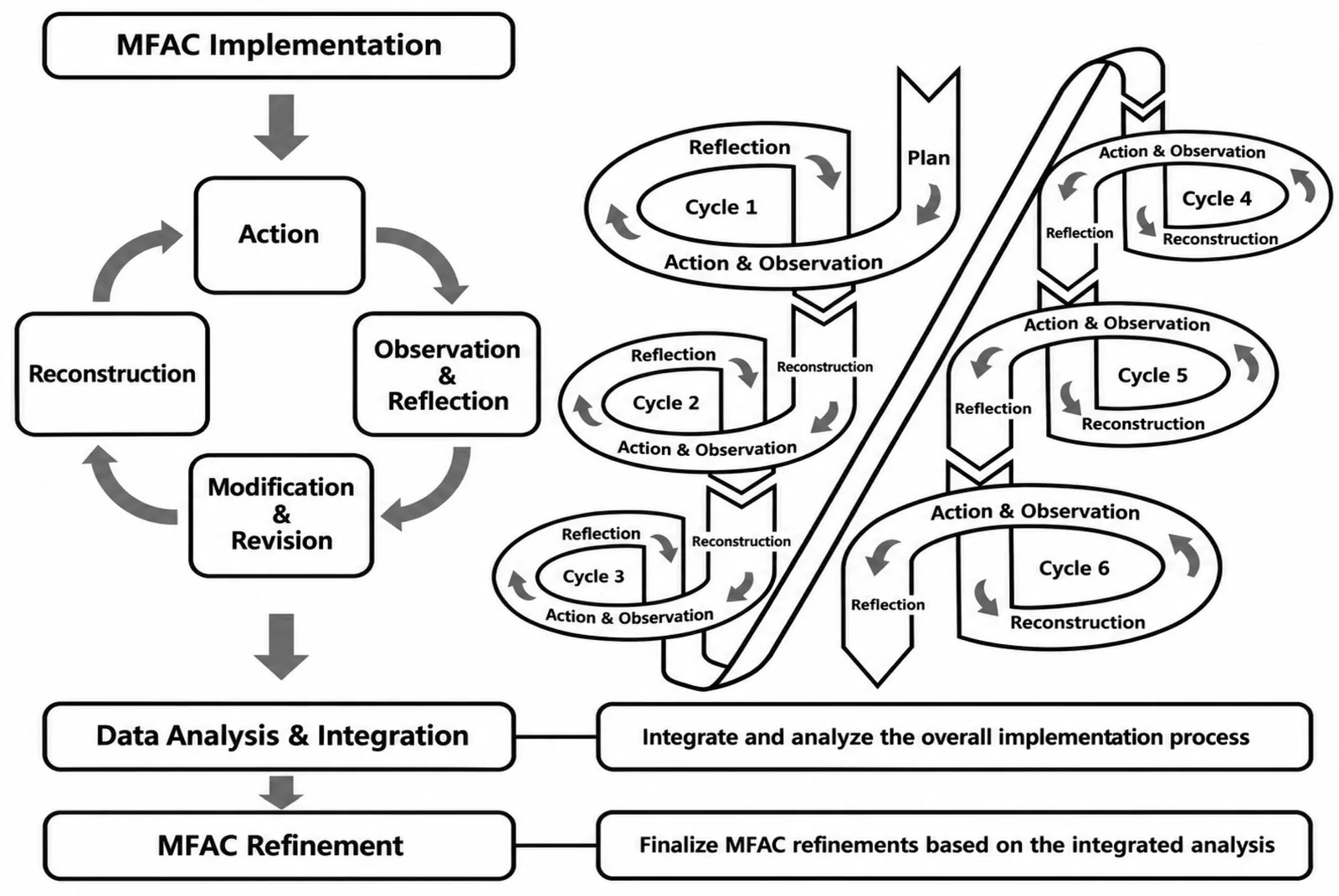
Overlapping and iterative participant session implementation structure of mindfulness–flow–awareness–coaching (MFAC). Note. Cycles 1–6 correspond to MFAC sessions 1–6; reflection and modification informed subsequent delivery.

Implementation followed a staggered and overlapping structure. Participants attended approximately weekly and progressed through Sessions 1–6 according to their individual schedules. Consequently, different participants could receive different sessions during the same period. Each participant session constituted an interconnected implementation and reflection unit rather than an independent action research study. Following each delivery, the first author reviewed participant records, observed responses, and researcher reflections. These reflections could inform the same participant’s subsequent session or a later delivery of the corresponding session to another participant. Figure 1 illustrates this iterative structure.

### 2.2. Participants, Recruitment, and Researcher Positioning

University students in Seoul and Gyeonggi Province, South Korea, responded to online and offline open recruitment calls from May to early August 2025. Sixteen individuals applied; Table 1 summarises the recruitment and selection process. The first author served as the researcher-facilitator and independently facilitated all MFAC sessions. The second author served as the research supervisor.

Eligible applicants were enrolled university students who wished to explore a personally identified concern, expressed interest in meditation and coaching, were willing to describe their experiences in detail, and were available to complete the full research procedure. The researchers considered previous experience with meditation or coaching during purposive selection but did not require such experience. Psychiatric diagnoses, current treatment status, and symptom severity were not formally screened during recruitment and were not used as eligibility criteria. The study recruited self-selected university student participants and was not designed to assess psychiatric conditions.

All six participants completed the study. One participant voluntarily continued after experiencing difficulty during Session 4 and attended an additional adjusted Session 4 one week later. Table 2 presents the participants’ characteristics and their primary reasons for participation.

We selected six participants as information-rich cases that enabled longitudinal within-case and cross-case analysis rather than statistical representation. The first author led the development of MFAC, which was informed by theoretical review, consultation with three specialists in meditation and counselling or coaching, and peer consultation with three colleagues. The first author independently facilitated all MFAC sessions. The second author served as the research supervisor.

### 2.3. Mindfulness–Flow-Awareness–Coaching Framework and Staggered Implementation

This study implemented MFAC through a flexible six-session framework integrating sensory-based mindfulness, reflective awareness, GROW-informed coaching dialogue, written reflection, and participant-selected between-session action activities. The initial framework drew on a theoretical review, peer consultation with three colleagues, and consultation with three specialists in meditation and counselling or coaching. The first author scheduled sessions according to participant availability, and sessions lasted approximately 100 min.

Coaching dialogue progressed from sensory noticing to emotional and contextual meaning and then to participant-selected action. Within MFAC, flow refers to continuity across sensory practice, reflection, coaching dialogue, and action rather than to Csikszentmihalyi’s (1990) optimal-experience construct or a measured psychological state. Table 3 presents the initial activities and time allocations, which the facilitator could adjust in response to participant needs.

Rather than using a fixed script, the facilitator selected and adapted questions according to participants’ sensory and emotional responses. Representative questions included: “What do you notice in your body?”; “What emotion, image, or memory does this sensory experience bring up?”; “How does this experience connect with a situation in your life now?”; and “What small action could you take to care for this need?” These questions supported a flexible progression from sensory noticing to contextual meaning-making and participant-selected action, without imposing a predetermined interpretation.

Participants attended approximately weekly and progressed through Sessions 1–6 according to their individual schedules. Because participants began MFAC at different times, implementation overlapped chronologically; earlier participants could attend Sessions 2 or 3 while later participants received Session 1. Sessions 1–6 were delivered sequentially within each participant, while participant schedules determined the chronological order of session deliveries across participants. Pungyo preceded Nabuteo in Sessions 1–3, whereas Nabuteo preceded Pungyo in Sessions 4–6.

Following each participant session, the first author reviewed participant records, observed responses, and researcher reflections. These reflections could inform the same participant’s subsequent session or a later delivery of the corresponding session to another participant. The facilitator could adjust the materials, duration, pacing, silence, questions, written activities, and action tasks. Participants could also pause, replace, or discontinue an activity.

The study included 36 planned participant sessions. One participant voluntarily attended an additional adjusted Session 4 one week after experiencing difficulty during the scheduled Session 4, bringing the total number of delivered sessions to 37.

### 2.4. Data Collection and Analysis

The first author collected data between August and December 2025 through six pre-MFAC interviews, 37 MFAC sessions, and six post-MFAC in-depth interviews, yielding 49 research encounters. Five participants completed six MFAC sessions each. One participant participated in seven session encounters because the initial Session 4 was discontinued following emotional overwhelm and was repeated in an adjusted form one week later. Pre-MFAC interviews explored participants’ concerns, reasons for participation, and prior experiences relevant to MFAC. Post-MFAC interviews examined experiences that participants described as memorable, difficult, limited, or meaningful. The dataset comprised audio and video recordings, verbatim transcripts, participant worksheets, sensory–emotion journals, action records, and the first author’s observational and reflexive notes. Quotations presented in this article were translated from Korean into English by the first author and lightly edited for readability without altering their intended meaning. The first author used video recordings to review nonverbal responses and session processes but did not code the video recordings as a separate data source.

The analysis proceeded at two related but analytically distinct levels. First, chronological implementation analysis examined how the researcher-facilitator implemented and refined MFAC across the 37 sessions. The first author organised materials by participant, date, and session to trace observed responses, implementation difficulties, safety concerns, proposed modifications, and their subsequent application, revision, or discontinuation. The first author retrospectively categorised changes that addressed an immediate participant need as individualised accommodations and changes retained in subsequent implementation as systematic refinements. Following each delivered session, the first author reviewed participant responses and possible modifications. We did not use a predetermined numerical threshold to distinguish changes. A change was classified as a systematic refinement when it was retained as an operational option or delivery principle in later deliveries. Representative chronological refinement sequences are presented in the Results section.

Second, reflexive thematic analysis, informed by Braun and Clarke (2006, 2019), examined patterns in participants’ reported experiences. Analysis involved repeated familiarisation with the data, primarily inductive coding informed by the research questions, within- and cross-case comparison, and the development and refinement of candidate themes. We treated theme generation as an interpretive process rather than as the identification of themes assumed to exist independently within the data. The analysis retained difficult, interrupted, limited, and ambiguous accounts to represent variation and avoid a uniformly positive interpretation.

The first author conducted all coding and the primary analysis. Through research supervision, the second author reviewed the coherence among the data, documented modifications, interpretations, and reflexive records but did not independently code or determine themes. The first author retained responsibility for the final analytic decisions.

The first author developed initial within-case codes from data extracts and organised them into provisional interpretive patterns. The first author then compared these codes and patterns across the six participants. The first author reviewed candidate themes against the full dataset, including accounts of discomfort, interruption, ambiguity, and incomplete action. During this process, the first author revised provisional formulations emphasising the expansion or formation of change to avoid implying uniform, enduring, or causally attributable changes. The final themes reflected greater openness to sensory and emotional experience, contextualised self-understanding through meaning-making, and movement towards feasible action. Table 4 provides illustrative examples of these analytic pathways.

### 2.5. Methodological Rigor, Reflexivity, and Ethical Considerations

The study supported methodological rigor through cross-source comparison, participant verification of transcript accuracy, chronological documentation, and reflexive notes. Neither author had a prior relationship with the participants. The first author used reflexive documentation, together with supervisory review, to consider potential influences arising from involvement in MFAC development and from the roles of facilitator and analyst.

The Institutional Review Board of Dongguk University approved the study (DUIRB2025-03-03; 2 April 2025). Participants provided written informed consent and received information about confidentiality, voluntary participation, withdrawal rights, and their right to pause, modify, or discontinue activities. The study used pseudonyms to protect confidentiality. Only the first and second authors had access to identifiable audio and video recordings. The authors stored the recordings on an encrypted external storage device and will retain them for five years in accordance with the Institutional Review Board (IRB)-approved data management protocol before permanently deleting them.

When an unexpected recollection left one participant emotionally overwhelmed during Session 4, the facilitator discontinued the session. No formal referral pathway was prearranged for the study; however, the facilitator discussed available professional support options with the participant and respected her decision regarding continuation. The study was not designed to provide clinical assessment or treatment. After the participant later voluntarily expressed a wish to continue, the facilitator reconfirmed her willingness to participate, and she attended an adjusted Session 4 one week later. She subsequently completed the study. The researcher treated her continuation as a participant-directed decision rather than as evidence that her emotional response had been resolved.

## 3. Results

The research questions provide the structure for the results. Section 3.1 presents patterns of iterative refinement identified through the implementation analysis. Section 3.2 presents themes generated through reflexive thematic analysis of participants’ accounts, including difficult, limited, and ambiguous experiences.

### 3.1. Patterns of Iterative Refinement in Mindfulness–Flow–Awareness–Coaching Delivery

Chronological implementation analysis identified how reflections on earlier deliveries informed subsequent deliveries of the corresponding sessions. The sensory sequence, mindfulness practice, coaching dialogue, written reflection, and between-session action activities remained consistent, while the facilitator adjusted duration, pacing, silence, sensory materials, questions, and action size. Table 5 presents representative refinement sequences across the six sessions.

Four practice-based principles emerged from these refinement patterns. First, sensory noticing preceded interpretation. Questions initially focused on bodily sensations, images, sounds, or other direct experiences before moving to emotional meaning, life context, and possible action. This sequence reduced pressure to provide an immediate explanation.

Second, psychological safety took priority over activity completion. The facilitator could adjust sensory intensity, duration, and channel, and participants could pause or discontinue activities. This principle became particularly evident when an unexpected recollection left Gyeoul emotionally overwhelmed during Session 4. The facilitator discontinued the scheduled session. After Gyeoul voluntarily decided to continue, she attended an additional adjusted Session 4 one week later.

Third, the facilitator balanced consistency in the core structure with responsive adaptation. The central MFAC components remained consistent, while the facilitator could adjust pacing, sensory materials, silence, question wording, and action size. This flexibility allowed the facilitator to accommodate immediate participant needs without treating every adjustment as a redevelopment of MFAC.

Fourth, the facilitator treated action activities as revisable attempts rather than performance requirements. When participants did not complete or discontinue actions, the facilitator reviewed them in relation to emotional states, practical constraints, and action burden. Participants could reduce, revise, or discontinue plans rather than evaluate them solely as successes or failures.

Together, these principles identify practice-based considerations for maintaining MFAC’s core structure while responding to sensory, emotional, procedural, and action-related demands.

### 3.2. Participants’ Reported Experiences of Mindfulness–Flow–Awareness–Coaching

Reflexive thematic analysis generated three themes: greater openness to sensory and emotional experience, contextualised self-understanding through meaning-making, and movement towards feasible action. These themes represent recurring patterns in participants’ accounts rather than uniform stages experienced by all participants. Table 4 illustrates how selected data extracts were developed into initial codes, cross-case interpretive patterns, and final themes. Table 6 summarises the themes and their interpretive boundaries.

#### 3.2.1. Greater Openness to Sensory and Emotional Experience

Participants commonly described a tendency to evaluate, control, or explain sensations and emotions before attending to them. They sometimes regarded tension, fatigue, distraction, pain, or weak sensation as obstacles to practice. In their accounts of MFAC, participants described moments when they attended to these responses as indications of their present condition, even when discomfort or anxiety remained.

Niryeonhwa expected the size of a singing bowl to correspond to the loudness of its sound. When her direct experience differed from this expectation, she reflected, “I realised that my way of thinking was not necessarily the right answer” (Niryeonhwa, Session 4). Her account suggests a momentary reconsideration of an existing assumption rather than a general change in her thinking style.

Byeori initially stated, “What is most difficult for me is not being able to predict what will happen” (Byeori, Session 1). During a later sound activity, she reported, “When I focused on the sound, my thoughts briefly disappeared” (Byeori, Session 4). This description represents a brief period of attention to sound rather than a resolution of her anxiety or need for predictability.

Other participants connected sensory and bodily responses with present concerns during coaching dialogue. Gyeoul related bodily pressure and the image of a “purple bruise” to fatigue and emotions suppressed while responding to others’ expectations. Isang associated pain, fatigue, and unpleasant sound with performance pressure and a need for recovery. Pungyo connected heartbeat and discomfort from a strong scent with emotional arousal in family relationships. Nabuteo associated coldness and weak sensation with emotions she had previously overlooked.

Sensory engagement was not uniformly positive. Strong or aversive scents, unpleasant sounds, environmental noise, unexpected recollections, distraction, and limited sensation sometimes interrupted participation. The facilitator shortened or adjusted some activities, changed the sensory channel, or discontinued the activity. Greater openness therefore refers to participants’ accounts of noticing sensory and emotional experiences, including discomfort and ambiguity, rather than to uniform calm or immersion.

#### 3.2.2. Contextualised Self-Understanding Through Meaning-Making

Participants used coaching dialogue to relate sensations, emotions, memories, and images to academic, career-related, interpersonal, and everyday contexts. They sometimes reconsidered reactions they had described as personal shortcomings, including perfectionism, anger, indecisiveness, and procrastination, in relation to needs, values, previous experiences, and situational demands. Because participants developed these meanings through coaching questions and interaction with the facilitator, we interpreted them as tentative and co-constructed.

In Session 3, Pungyo described one fragrance as strong enough to make him feel dizzy. When the facilitator asked, “What message do you think this scent is giving you?” Pungyo replied, “A momentary stimulus does not determine everything.” He subsequently connected the scent and his reaction to it with impulsive speech during conflict with his parents. This exchange illustrates how sensory discomfort became a starting point for reflecting on his emotional responses within a family relationship.

In Session 3, after Nabuteo associated a preferred scent with comfort, the facilitator asked, “What could you do, within what is possible for you now, to feel more at ease?” Nabuteo replied, “If I start a task or report with a deadline and make some progress, I think I would feel less anxious.” At the beginning of Session 4, she reported, “I had kept putting off sending an email to my professor, but after applying the scent, I did it, and it was quite okay.” This sequence linked sensory self-support with a manageable and self-directed way of initiating action.

Isang related his responses to ambiguous visual material, unpleasant sounds, and tea served in different cups to his discomfort with uncertainty and concern with achievement and external recognition. He described one realisation as “a feeling of being pierced through” and considered his achievement orientation as a source of pressure and a motivation for development. His account illustrated how he interpreted sensory experiences in relation to achievement and uncertainty during coaching dialogue.

Niryeonhwa related dichotomous thinking to her search for certainty. Byeori compared her context-dependent sense of self to “a liquid that changes shape according to its container” and considered whether this might represent adaptability rather than inconsistency. Gyeoul questioned whether becoming speechless during one presentation represented her overall ability. Nabuteo described procrastination as an “overprotective” response to anticipated failure and expanding demands. These accounts illustrate how participants reconsidered prior self-evaluations within their life contexts.

These interpretations did not simply replace negative self-evaluations with positive explanations. Some remained tentative, and participants did not describe every sensory activity as meaningful or useful. The theme therefore captures the contextual and sometimes tentative meanings that participants articulated through coaching dialogue.

#### 3.2.3. Movement Towards Feasible Action

Participants described how high standards, anticipated evaluation, and a desire for certainty could delay action. Their accounts included selecting, attempting, reviewing, revising, and discontinuing actions according to their circumstances.

Nabuteo, who tended to postpone tasks with uncertain or potentially complicated outcomes, articulated the principle, “Start first, and if it does not work, think again at that point.” In the final session, she contacted a potential officer for a student organisation rather than continuing to deliberate about possible difficulties. This action represented a situated example of initiating a bounded action. Her earlier account of sending an email to a professor after using a preferred scent similarly illustrated a small, self-selected attempt to begin a postponed task.

Niryeonhwa divided decisions concerning her career and double major into smaller steps, including reading relevant materials and adjusting her schedule. She summarised her approach as follows: “Rather than comparing myself with others, I will do as much as I can.” Byeori selected an external accountability arrangement, while Isang identified actions within his immediate control and considered whether rest could support his academic engagement. Gyeoul used brief meditation and activity records, and Pungyo selected time-outs and greater relational distance during conflict. These actions differed in form and reflected each participant’s circumstances rather than a common behavioural outcome.

Participants did not complete all actions as planned. They interrupted, reduced, revised, or discontinued some actions in response to emotional states and everyday constraints. These variations indicate that feasible action involves initiating an activity and adjusting or discontinuing it when circumstances change. The theme therefore captures participants’ situated and revisable engagement with action.

Across the three themes, participants’ accounts included openness as well as discomfort, meaningful interpretations as well as uncertainty, and attempted actions as well as revision or discontinuation. These themes represent patterned accounts of participation rather than a uniform sequence of change or evidence of MFAC effectiveness.

## 4. Discussion

This study analysed the iterative refinement of MFAC and participants’ experiences of participation. The implementation analysis distinguished individualised accommodations addressing immediate participant needs from systematic refinements retained in subsequent delivery. Although the facilitator maintained the core structure, they adjusted sensory materials, pacing, questions, and action activities in response to participants’ sensory reactions, emotional states, and action burden. Because the analysis identified this distinction retrospectively, it represents an analytical interpretation of the implementation process rather than a fixed attribute of the MFAC framework.

Participants described moments of attending to bodily and emotional responses before evaluating or explaining them. These accounts are consistent with mindfulness perspectives on less judgmental attention and embodied approaches emphasising relationships among bodily states, environments, and meaning (Bishop et al., 2004; Farb et al., 2015; Wilson, 2002). However, participants did not find sensory engagement uniformly helpful. Discomfort, weak sensation, distraction, unexpected recollections, and interrupted activities suggest that sensory awareness functioned as a context-dependent entry point for reflection rather than an inherently beneficial process. The adjusted repetition of Session 4 further highlighted the importance of psychological safety, participant choice, and responsive adaptation.

Coaching dialogue provided a context in which participants related sensory and emotional experiences to academic, career-related, interpersonal, and everyday concerns. This finding is broadly consistent with studies that integrate mindfulness and coaching (Collard & Walsh, 2008; Corti & Gelati, 2020; Spence et al., 2008). Nevertheless, participants and the facilitator co-constructed these interpretations through their interactions, which occurred within Korean university contexts involving academic performance, family expectations, social comparison, and sensitivity to evaluation. Because the study did not examine cultural influences comparatively, readers should not regard these patterns as uniquely Korean or assume they apply similarly across cultural settings.

Across participants, concerns related to evaluation, comparison, family relationships, and future planning appeared in different thematic forms. Byeori’s self-evaluation was associated with difficulty remaining with ambiguity, Nabuteo linked anticipated failure and comparison with delayed action, and Pungyo connected emotional arousal with family conflict. These observations do not establish culturally specific mechanisms, but show that sensory noticing, meaning-making, and action planning were interpreted within social contexts in which achievement, evaluation, and family relationships were salient.

Responsive adaptation was bounded by the core sequence of sensory noticing, reflective coaching dialogue, written reflection, and participant-selected action. For example, when tea preparation divided attention, the facilitator prepared and served the tea rather than removing the taste-based activity; when distress made continuation inappropriate, safety and participant choice took priority over activity completion. Thus, adaptation modified delivery while preserving the framework’s core structure rather than becoming unrestricted individualisation.

Participants also described selecting smaller actions, using available support, and revising plans according to their circumstances. These accounts reflect the importance of action feasibility and context (Albarracín et al., 2024). However, MFAC explicitly incorporated action planning, and participants left some activities incomplete or reduced or discontinued them. Therefore, the findings concern participants’ situated engagement with action rather than the development of a general or sustained capacity for self-regulation.

Therefore, the findings characterise MFAC as a practice-informed, adaptable framework that integrates sensory-based mindfulness, reflective coaching dialogue, and revisable action activities. The study identified implementation considerations involving sensory preference, emotional activation, psychological safety, question timing, and action burden. These constitute practice-based implementation considerations rather than evidence of effectiveness or superiority over other approaches.

## 5. Limitations and Future Directions

The small, self-selected sample of six Korean university students, individualised delivery, and absence of comparison conditions, baseline behavioural data, standardised outcome measures, and follow-up assessment limit the findings. Participants’ interest in meditation and coaching may have influenced their engagement, and we cannot generalise the findings to broader student or clinical populations. Because the facilitator adapted the intervention during implementation, participants shared a core framework but did not receive fully identical delivery.

The first author’s involvement in MFAC development, together with the roles of facilitator and analyst, increased the possibility of researcher influence, expectancy effects, social desirability, and co-constructed interpretations. Participants may also have shaped their accounts or selected actions in ways they believed were expected by the facilitator or the study, creating possible demand characteristics in addition to social desirability. Because MFAC combined sensory activities, coaching dialogue, written reflection, and an ongoing facilitator–participant relationship, the present design cannot disentangle the contribution of MFAC-specific components from that of the coaching relationship or repeated research contact. Reflexive documentation, transcript verification, and research supervision supported transparency but could not eliminate these influences. Future studies should prospectively document adaptations, involve independent facilitators and analysts, include participants from diverse educational and cultural contexts, and conduct longer-term follow-up. Researchers would need comparative designs and appropriate outcome measures to evaluate effectiveness or sustained change.

## 6. Conclusions

This exploratory qualitative action research contributes to understanding MFAC as a context-responsive process rather than a fixed intervention delivered uniformly across participants. Its iterative implementation showed that maintaining a consistent core structure need not preclude adjustments to sensory materials, pacing, dialogue, and action activities. Such responsiveness became particularly important when sensory engagement generated discomfort, emotional activation, or action burden.

The findings also suggest that sensory noticing, meaning-making through coaching dialogue, and feasible action were interrelated but not uniformly progressive experiences. Participants’ accounts included increased openness and contextualised understanding alongside ambiguity, interruption, and discontinued action. This complexity indicates that researchers and practitioners should not assume that sensory engagement is inherently beneficial and should consider psychological safety, participant choice, and revisability central to MFAC delivery.

Therefore, the findings position MFAC as a practice-informed framework for integrating sensory-based mindfulness with reflective dialogue and action planning. However, the present findings establish implementation knowledge rather than effectiveness or sustained psychological change. Further research should examine its applicability across facilitators, populations, and cultural contexts and evaluate outcomes through comparative and longitudinal designs.

## Figures and Tables

**Table 1:** Participant recruitment and selection.

Stage	Procedure and Criteria	Outcome
Open recruitment	Online and offline voluntary applications	16 applicants
Eligibility review	University enrolment, personally identified concerns, interest in meditation and coaching, willingness to describe experiences, and availability	9 applicants
Final selection	Relevance to the research questions, potential to provide detailed experiential accounts, previous meditation or coaching experience where applicable, and availability for the full procedure	6 participants

**Table 2:** Participant characteristics.

Participant	Age/Gender	Major and Year	Primary Reason for Participation
Niryeonhwa	24/F	Psychology, third year	Self-understanding and career exploration
Byeori	23/F	Software convergence, fourth year	Perfectionistic concerns and emotional recovery
Isang	19/M	Interdisciplinary engineering, first year	University adjustment and self-expression
Gyeoul	23/F	Architecture, fourth year	Future anxiety and self-acceptance
Pungyo	19/M	Computer software, first year	Emotional awareness and self-understanding
Nabuteo	22/F	Interdisciplinary studies (law), third year	Reducing self-comparison and exploring career options

Note. All names are Korean pseudonyms presented in their Romanised form.

**Table 3:** Initial framework for the six MFAC sessions.

Session	Mindfulness Activity	Awareness Focus	Coaching and Action Focus	Initial Time Allocation (Min)
1. Touch	Breathing, body scan, and tactile awareness	Present bodily sensations and emotions	Goal–Reality: exploring the present self; sensation record	15/25/30/15/15
2. Vision	Painting observation and visual expression	Personal values, strengths, and perspectives	Reality–Options: self-understanding and alternative perspectives; visual expression	10/25/40/15/10
3. Smell	Breathing and aroma meditation	Sensations, emotions, and memories	Reality–Options: emotional exploration and acceptance; scent–emotion record	10/25/35/20/10
4. Hearing	Breathing, singing bowls, and nature sounds	Inner responses, self-talk, and needs	Reality–Options: self-expression and self-understanding; self-supporting statement	10/30/30/20/10
5. Taste	Breathing and mindful tea drinking	Personal needs, choices, and life context	Options–Will: direction and one feasible action	10/30/35/15/10
6. Multisensory integration	Integrated sensory activities	Integrating sensory experience and self-reflection	Reality–Options–Will: values, direction, and a revisable action plan	10/35/30/15/10

Note. Times correspond to opening and review, mindfulness activity, coaching dialogue, written reflection and action planning, and closing. Flow refers to continuity across these components, and the facilitator could adjust time allocations according to participant responses.

**Table 4:** Illustrative analytic pathways from data extracts to final themes.

Data Extract	Initial Code	Cross-Case Interpretive Pattern	Final Theme
“When I stay still, I think I can feel more.” (Niryeonhwa, Session 1)	Discovering bodily sensations through pausing	Attending to bodily and emotional states before evaluation	Greater openness to sensory and emotional experience
“When I focused on the sound, my thoughts briefly disappeared.” (Byeori, Session 4)	Brief attentional engagement with sound	Using sensory attention to notice the present experience	Greater openness to sensory and emotional experience
“Avoiding it seems to make me feel somewhat better, both mentally and physically.” (Gyeoul, Session 3)	Recognising the short-term protective function of avoidance	Reconsidering avoidance in relation to emotional burden and context	Contextualised self-understanding through meaning-making
“A liquid that changes shape according to its container.” (Byeori, Session 2)	Representing the context-dependent self through metaphor	Reinterpreting situational self-adjustment as potentially adaptive	Contextualised self-understanding through meaning-making
“Start first, and if it does not work, think again at that point.” (Nabuteo, Session 6)	Initiating action despite uncertainty	Selecting a bounded and revisable action	Movement towards feasible action
Nabuteo requested that her action be checked every two days. (Session 5)	Using external accountability to support action	Selecting participant-chosen environmental support	Movement towards feasible action
An unexpected recollection led to emotional overwhelm and discontinuation of the session. (Gyeoul, Session 4)	Emotional overwhelm during sensory activity	Recognising the limits and potential difficulty of sensory engagement	Greater openness to sensory and emotional experience (divergent case)

Note. Examples illustrate selected analytic pathways, including divergent evidence, rather than an exhaustive codebook.

**Table 5:** Representative chronological refinement sequences in mindfulness–flow–awareness–coaching delivery.

Session	Chronological Observation and Response	Retained Refinement
1. Touch	Niryeonhwa: Long body scan reduced engagement → Byeori: Duration adjusted and uncomfortable sensations explicitly permitted → Isang and Gyeoul: Facilitator explanation reduced and transition questions strengthened	Flexible duration, participant-centered questions, and attention to nonverbal responses
2. Vision	Niryeonhwa: Detailed visual analysis generated fatigue → Byeori: Viewing distance adjusted and noninterpretive observation encouraged → Later deliveries: More silence and feeling- or metaphor-based prompts	Observation before explanation and reduced interpretive pressure
3. Smell	Niryeonhwa: Suggested exploring unpleasant scents → Pungyo: Strong scent caused dizziness → Nabuteo: Uncomfortable scent replaced with peppermint	Participant choice to reduce, replace, or discontinue scents
4. Hearing	Niryeonhwa: Emotional memories competed with attention to sound → Byeori: Lower tones and additional silence were used → Isang: Sensation-focused guidance reduced analytical explanation → Gyeoul: An unexpected recollection led to session discontinuation → Nabuteo: Participant-selected music was used → Pungyo: An aversive rain sound was replaced with preferred game music	Flexible pitch and sound selection, activity pausing, sensory channel change, and safety prioritisation
5. Taste	Niryeonhwa: Preparing tea divided attention → Byeori and later participants: Facilitator prepared and served tea	Preparation and reduced procedural burden
6. Multisensory integration	Across deliveries: Broad plans, incomplete activities, fatigue, and uncertainty increased action burden → Plans were reduced or revised according to participant circumstances	One feasible, participant-selected, and revisable action

Note. All 37 delivered sessions were reviewed in the chronological implementation analysis. Because adjustments were retrospectively identified from implementation records rather than prospectively coded as discrete, mutually exclusive events, the table presents representative refinement sequences rather than frequency counts. Arrows indicate how an earlier observation informed a subsequent delivery. Systematic refinements were changes that were retained as operational options or delivery principles in later sessions.

**Table 6:** Themes and interpretive boundaries in participants’ mindfulness–flow–awareness–coaching experiences.

Theme	Patterned Participant Accounts	Interpretive Boundaries and Variations
Greater openness to sensory and emotional experience	Attending to sensations before evaluation and noticing bodily or emotional responses	Strong scents, unpleasant sounds, distraction, weak sensation, and emotional discomfort sometimes led to adjustment, pausing, or discontinuation.
Contextualised self-understanding through meaning-making	Relating sensations, emotions, images, and memories to personal and everyday contexts	Meanings were tentative and co-constructed through coaching dialogue; not every activity was described as meaningful.
Movement towards feasible action	Selecting smaller actions and reviewing or revising attempted actions	Action planning was part of MFAC; completion did not indicate a general capacity for self-regulation, and some actions were reduced or discontinued.

## Data Availability

The data presented in this study are available on request from the corresponding author. The data are not publicly available due to privacy and ethical restrictions related to the confidentiality of participants’ qualitative interview and session data.
